# Global Expansion of Pacific Northwest *Vibrio parahaemolyticus* Sequence Type 36

**DOI:** 10.3201/eid2602.190362

**Published:** 2020-02

**Authors:** Michel Abanto, Ronnie G. Gavilan, Craig Baker-Austin, Narjol Gonzalez-Escalona, Jaime Martinez-Urtaza

**Affiliations:** University of La Frontera, Temuco, Chile (M. Abanto);; Instituto Nacional de Salud, Lima, Peru (R.G. Gavilan);; The Centre for Environment, Fisheries and Aquaculture Science, Weymouth, UK (C. Baker-Austin, J. Martinez-Urtaza);; US Food and Drug Administration, College Park, Maryland, USA (N. Gonzalez-Escalona)

**Keywords:** foodborne pathogens, outbreak, genomic epidemiology, Vibrio infections, bacteria, ST36, sequence type 36, Pacific Northwest, *Vibrio parahaemolyticus*, global expansion, epidemiology, El Niño, marine bacteria, phylogenetic analysis, food safety, Canada, United States, North America, Spain, Peru

## Abstract

We report transcontinental expansion of *Vibrio parahaemolyticus* sequence type 36 into Lima, Peru. From national collections, we identified 7 isolates from 2 different Pacific Northwest complex lineages that surfaced during 2011–2016. Sequence type 36 is likely established in environmental reservoirs. Systematic surveillance enabled detection of these epidemic isolates.

Compared with other major foodborne illnesses, *Vibrio parahaemolyticus* infections have been steadily increasing (*1*); thus, *V*. *parahaemolyticus* has become the leading cause of seafood-related bacterial infections globally. The US Centers for Disease Control and Prevention estimated that the average annual incidence of all *Vibrio* infections increased by 54% during 2006–2017 (*2*), and *V*. *parahaemolyticus* infections were responsible for a large percentage of this increase in the later years (*3*). *V*. *parahaemolyticus* is believed to be responsible for ≈35,000 human infections each year in the United States alone (*4*) and has been identified as the leading cause of foodborne infections in China since the 1990s (*5*).

In some areas of the world, the steady increase in local numbers of cases associated with *V*. *parahaemolyticus* has coincided with the overall geographic expansion of *V*. *parahaemolyticus* disease. *V*. *parahaemolyticus* cases are now being regularly reported in areas with little previous incidence, including South America and northern Europe (*6*,*7*). Although the precise circumstances and factors driving the growth in case numbers are unclear, the transition of *V*. *parahaemolyticus* disease from a regional to a more global pathogen has been directly connected with the emergence of isolates with epidemic potential.

*V*. *parahaemolyticus* and *V*. *cholerae* represent the only 2 documented instances of global expansion of human pathogenic marine bacteria (*8*). Pandemic *V*. *cholerae* emerged >50 years ago, and intercontinental expansion of *V*. *parahaemolyticus* began ≈25 years ago. *V*. *parahaemolyticus* sequence type (ST) 3 emerged in India in 1996 and rapidly underwent transcontinental dissemination, reaching almost all continents (*9*). ST3 causes infections worldwide and persists as the dominant type in some countries of Asia, including China (*5*).

ST3 was the only known example of *V*. *parahaemolyticus* transcontinental expansion until 2012, when a new type, ST36, was identified outside its endemic region (the Pacific Northwest of North America) (*10*). ST36 infections were initially reported in the northeastern United States, increasing the number of *V*. *parahaemolyticus* infections in this region (*3*). A few months later, they were reported in a single large outbreak in Spain (*11*). An in-depth genomewide analysis of representative isolates from the Pacific Northwest, northeastern United States, and Spain showed that ST36 is a highly dynamic population and that the *V*. *parahaemolyticus* strains causing infections in the northeastern United States had diverged from the original lineage in the Pacific Northwest over the course of the cross-continent eastward expansion (*12*). The strains in the northeastern United States and Spain arose from 2 distinctive ST36 populations. Although the international spread of this population is a concern, ST36 has not been documented outside of the United States since the outbreak in Spain in 2012 (*11*).

## The Study

After the emergence of cholera in 1991 in Peru and reemergence in 1998, both concurrent with El Niño events, the Peruvian National Institute of Health (Lima, Peru) implemented a contingency plan for preparedness to respond to every El Niño episode. This contingency plan involves intensive investigations of all *Vibrio* isolates acquired in clinical settings and enhanced monitoring of environmental sources. Among the characterized *V. parahaemolyticus* isolates obtained over the course of surveillance, we identified 5 clinical ST36 isolates (3 in 2015 and 2 in 2016) from Lima (Table). After reviewing the *V. parahaemolyticus* isolates deposited in the historical collection of the Peruvian National Institute of Health over the past 30 years, we were able to identify 2 additional isolates (1 from a seawater sample collected in 2011 and 1 from a clinic in 2012).

**Table:** Sequence type 36 isolates identified in clinical settings and from environmental sources, Lima, Peru, 2011–2016

Isolate	Alias	Year	Source
CFSAN062371	3.369–15	2011	Environmental
CFSAN062362	1.004–13	2012	Clinical
CFSAN062300	3.252–16	2015	Clinical
CFSAN062366	1.147–15	2015	Clinical
CFSAN062373	1.146–15	2015	Clinical
CFSAN062273	1.210–16	2016	Clinical
CFSAN062350	1.166–15	2016	Clinical

**Figure 1:**
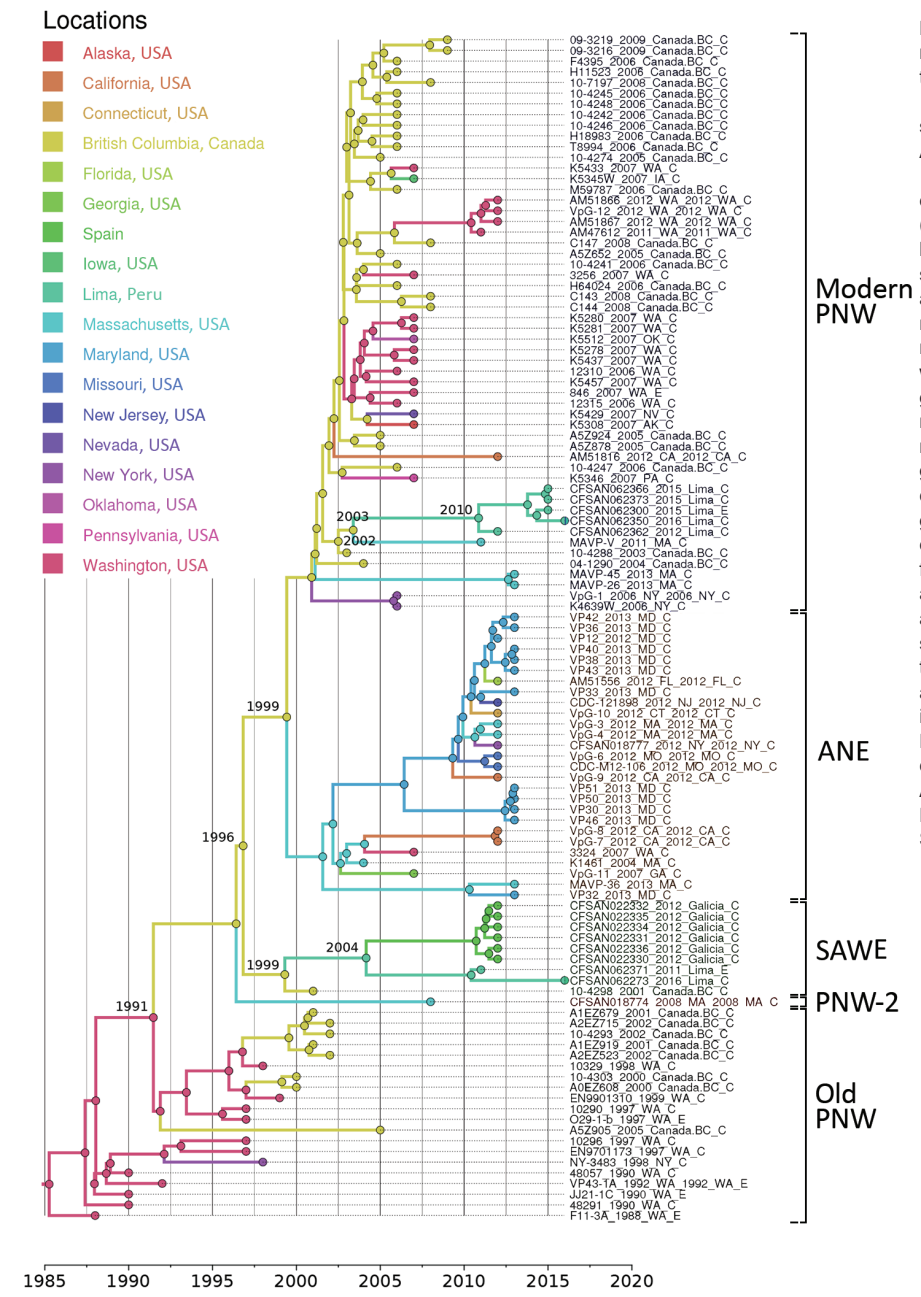
Phylogenetic reconstruction of transcontinental spread of *Vibrio parahaemolyticus* sequence type 36, North America, Peru, and Spain, 1985–2016. Timeline was estimated with BEAST (https://beast.community) by applying a Bayesian skyline demographic model and uncorrelated lognormal molecular clock. Single-nucleotide polymorphisms were identified in core genomes after the removal of recombination. Branch colors represent the most probable geographic origin of the last common ancestor of the group. Dates at nodes show estimated divergence dates from most recent common ancestor. Old PNW is the ancestral population (last strain identified in 2002) of the PNW lineage complex, and the modern PNW lineage is the currently circulating PNW population. *Vibrio* classifications are indicated. ANE, Atlantic Northeast; PNW, Pacific Northwest; SAWE, South America–West Europe.

**Figure 2:**
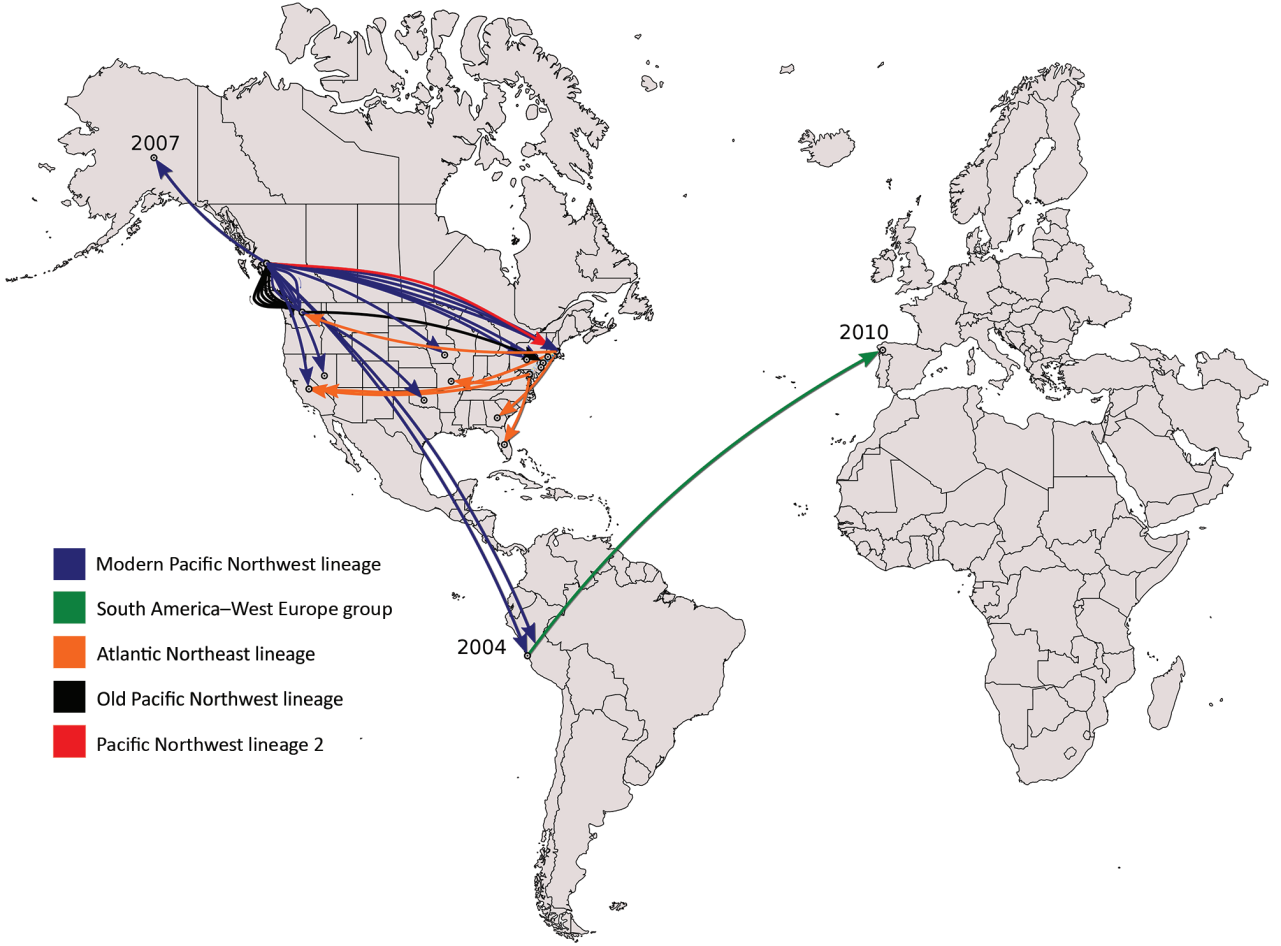
Transcontinental spread of *Vibrio parahaemolyticus* sequence type 36, North America, Peru, and Spain, 1985–2016. Timeline was estimated by using BEAST (Bayesian evolutionary analysis by sampling trees). Years on map indicate the inferred dates of arrival of *V*. *parahaemolyticus* sequence type 36 to that country. Old Pacific Northwest is the ancestral population (last strain identified in 2002) of the Pacific Northwest lineage complex, which also includes the modern (i.e., currently circulating) Pacific Northwest lineage, Pacific Northwest lineage 2, Atlantic Northeast lineage, and the South America–West Europe group.

We performed a genomewide phylogenetic analysis of a global collection of 111 ST36 isolates (Appendix Table) obtained during the past 30 years from the United States (west and east coasts), Canada, Spain, and Peru. Results indicated that the isolates from Peru were of 2 different genetic variants (Figures 1, 2): 5 clustered with the modern (i.e., currently circulating) Pacific Northwest lineage, and 2 clustered in a distinctive group comprising isolates from the 2012 outbreak in Spain. Analysis of the phylodynamics of transmission by Bayesian inference suggested the existence of 2 independent and almost contemporary introductions of ST36 into Peru in 2011, both originating from 2 distinct modern Pacific Northwest variants. The group comprising isolates from Peru and Spain appeared to have diverged from a common ancestor around 2004. Considering the number of years from the last common ancestor of both Peru lineages and that other closely related genetic variants are absent from the dataset, intermediary populations might exist in regions not yet scrutinized.

The identification of ST36 in Peru provides additional evidence of the extraordinary dynamics of *Vibrio* infections in this region. Since the emergence of cholera in 1991 and the subsequent implementation of an active surveillance system for *Vibrio* diseases in Peru, several instances of emergence of major epidemic clones of *V*. *parahaemolyticus* have been reported in the country. Although the sources and routes of introduction of these foreign clones remain yet undetermined, a growing body of evidence has linked the epidemic dynamics and spreading of disease in this particular region of South America to El Niño (*13*). During the past 30 years, the emergence of cases in Peru associated with new clones of *Vibrio* has been sharply influenced by the onset of El Niño conditions, which has also shaped the extent and severity of epidemics (*14*,*15*). The arrival of extraordinary weather conditions brought on by El Niño (i.e., a combination of heavy rains and heat waves) provides the ideal conditions for the proliferation of *Vibrio* spp. in the environment. These circumstances, along with disruption of sanitary infrastructure caused by floods and landslides, can help generate the perfect conditions for the explosive emergence of *Vibrio* diseases.

Despite the evidence connecting the epidemiology of *Vibrio* in Peru to El Niño, little is known about the mechanisms of global dispersal and introduction of foreign epidemic clones into the region. Ballast water from cargo ships and marine heat waves have been associated with some instances of disease emergence elsewhere (*12*). Another mechanism that might be involved in the dispersal of *V*. *parahaemolyticus* populations is the international trade of shellfish, which was suggested to facilitate the introduction of ST36 into the United States and Spain (*12*).

## Conclusions

We report the transcontinental expansion of ST36 *V*. *parahaemolyticus* into South America. The presence of ST36 in clinical and environmental settings in Peru emphasizes the exceptional epidemic potential of the Pacific Northwest complex and *V. parahaemolyticus* as a human pathogen. The long-term persistence and presence of environmental isolates suggest the successful establishment of ST36 in environmental reservoirs. ST36’s ability for intercontinental dispersal, along with its highly pathogenic nature (*1*), make this *Vibrio* population a major public health concern. Furthermore, Peru has shown that implementation of systematic surveillance for *Vibrio* species can facilitate the detection of emerging transnational epidemic strains. This strategy will play a crucial role under exceptional climatic conditions, such as those generated by El Niño, where enhanced risk for outbreaks is likely.

Appendix More information about global expansion of Pacific Northwest *Vibrio parahaemolyticus* sequence type 36.
