# Supplementary material for: Global Expansion of Pacific Northwest Vibrio parahaemolyticus Sequence Type 36
Source: Emerg Infect Dis. 2020 Feb;26(2):323–6. doi: 10.3201/eid2602.190362 (PMC6986845; doi:10.3201/eid2602.190362)
Supplement: Appendix — More information about global expansion of Pacific Northwest Vibrio parahaemolyticus sequence type 36. [file 19-0362-Techapp-s1.pdf]

Article DOI: https://doi.org/10.3201/eid2602.190362

Global Expansion of Pacific Northwest *Vibrio parahaemolyticus* Sequence Type 36

Appendix

Appendix Table. List of *Vibrio parahaemolyticus* ST36 strains used in this study and their metadata

Strain	Alias	Province or city	Country	Year	Source	Accession No.
09–3219	09–3219	British Columbia	Canada	2009	Clinical	NZ_LRSW01000000
09–3216	09–3216	British Columbia	Canada	2009	Clinical	JXVJ00000000
F4395	F4395	British Columbia	Canada	2006	Clinical	NZ_LRFU01000000
H11523	H11523	British Columbia	Canada	2006	Clinical	LRFY01000000
10–7197	10–7197	British Columbia	Canada	2008	Clinical	JXUX00000000
10–4245	10–4245	British Columbia	Canada	2006	Clinical	JXVG00000000
10–4248	10–4248	British Columbia	Canada	2006	Clinical	JXVD00000000
10–4242	10–4242	British Columbia	Canada	2006	Clinical	JXVH00000000
10–4246	10–4246	British Columbia	Canada	2006	Clinical	JXVF00000000
H18983	H18983	British Columbia	Canada	2006	Clinical	NZ_LRST00000000
T8994	T8994	British Columbia	Canada	2006	Clinical	NZ_LRGA01000000
10–4274	10–4274	British Columbia	Canada	2005	Clinical	JXVC00000000
K5433	CDC_K5433	Washington	USA	2007	Clinical	NZ_MIUR01000000
K5345W	CDC_K5345W	Iowa	USA	2007	Clinical	NZ_MIUN01000000
M59787	M59787	British Columbia	Canada	2006	Clinical	LRJZ01000000
AM51866	CDC4967	Washington	USA	2012	Clinical	LHRM00000000
VpG-12	CDC4902	Washington	USA	2012	Clinical	LHRG00000000
AM51867	CDC4970	Washington	USA	2012	Clinical	LHRN00000000
AM47612	CDCAM47612	Washington	USA	2011	Clinical	LHRI00000000
C147	C147	British Columbia	Canada	2008	Clinical	LPVM01000000
A5Z652	A5Z652	British Columbia	Canada	2005	Clinical	LQCE01000000
10–4241	10–4241	British Columbia	Canada	2006	Clinical	JXVI00000000
3256	3256	Washington	USA	2007	Clinical	AZGS00000000
H64024	H64024	British Columbia	Canada	2006	Clinical	LRFZ01000000
C143	C143	British Columbia	Canada	2008	Clinical	LPVB01000000
C144	C144	British Columbia	Canada	2008	Clinical	LPVC01000000
K5280	CDCK5280	Washington	USA	2007	Clinical	LHQX00000000
K5281	CDC_K5281	Washington	USA	2007	Clinical	NZ_MIUB01000000
K5512	CDC_K5512	Oklahoma	USA	2007	Clinical	NZ_MIUZ01000000
K5278	CDC_K5278	Washington	USA	2007	Clinical	NZ_MITY01000000
K5437	CDC_K5437	Washington	USA	2007	Clinical	NZ_MIUT01000000
12310	12310	Washington	USA	2006	Clinical	AYXP00000000
K5457	CDC_K5457	Washington	USA	2007	Clinical	NZ_MIUX01000000
846	846	Washington	USA	2007	Environmental	NZ_AOOX00000000
12315	12315	Washington	USA	2006	Clinical	NZ_AOPF00000000
K5429	CDC_K5429	Nevada	USA	2007	Clinical	NZ_MIUQ01000000
K5308	CDC_K5308	Alaska	USA	2007	Clinical	NZ_MIUE01000000
A5Z924	A5Z924	British Columbia	Canada	2005	Clinical	LQCV01000000
A5Z878	A5Z878	British Columbia	Canada	2005	Clinical	NZ_LQCT00000000
AM51816	CDC_M12X03280	California	USA	2012	Clinical	LHRL00000000
10–4247	10–4247	British Columbia	Canada	2006	Clinical	JXVE01000000
K5346	CDC_K5346	Pennsylvania	USA	2007	Clinical	NZ_MIUO00000000
CFSAN062366	1.147–15	Lima	Peru	2015	Clinical	SRR8103881
CFSAN062373	1.146–15	Lima	Peru	2015	Clinical	SRR8103882
CFSAN062300	3.252–16	Lima	Peru	2015	Clinical	SRR8103883
CFSAN062350	1.166–15	Lima	Peru	2016	Clinical	SRR8103885
CFSAN062362	1.004-13	Lima	Peru	2012	Clinical	WSRX00000000
MAVP-V	MAVP-V	Massachusetts	USA	2011	Clinical	NZ_LBHO00000000
10–4288	10–4288	British Columbia	Canada	2003	Clinical	JXVB00000000
04–1290	04–1290	British Columbia	Canada	2004	Clinical	NZ_JXVK01000000
MAVP-45	MAVP-45	Massachusetts	USA	2013	Clinical	NZ_LBHN01000000
MAVP-26	MAVP-26	Massachusetts	USA	2013	Clinical	NZ_LBHD01000000
VpG-1	CDCK4639–1	New York	USA	2006	Clinical	LHRH00000000
K4639W	CDC_K4639W	New York	USA	2006	Clinical	NZ_MITA00000000

Strain	Alias	Province or city	Country	Year	Source	Accession No.
VP42	CFSAN007460	Maryland	USA	2013	Clinical	JNTS02000000
VP36	CFSAN006133	Maryland	USA	2013	Clinical	JNTP02000000
VP12	CFSAN006129	Maryland	USA	2012	Clinical	JNTM02000000
VP40	CFSAN006135	Maryland	USA	2013	Clinical	JNTR02000000
VP38	CFSAN006134	Maryland	USA	2013	Clinical	JNTQ02000000
VP43	CFSAN007461	Maryland	USA	2013	Clinical	JNTT02000000
AM51556	CDC_JBI 12000789	Florida	USA	2012	Clinical	LHRK00000000
VP33	CFSAN006132	Maryland	USA	2013	Clinical	JNTO02000000
CDC-121898	CFSAN018775	New Jersey	USA	2012	Clinical	LHRP00000000
VpG-10	CT_220206001	Connecticut	USA	2012	Clinical	LHRE00000000
VpG-3	MA_12EN2941	Massachusetts	USA	2012	Clinical	LHRC00000000
VpG-4	MA_12EN2945	Massachusetts	USA	2012	Clinical	LHRD00000000
CFSAN018777	CDC101325304	New York	USA	2012	Clinical	LHRQ00000000
VpG-6	CFSAN001597	Missouri	USA	2012	Clinical	LHRH01000000
CDC-M12–106	CDC_M12–108 G	Missouri	USA	2012	Clinical	LHQZ00000000
VpG-9	CA_M12X02735	California	USA	2012	Clinical	LHRF00000000
VP51	CFSAN026730	Maryland	USA	2013	Clinical	NZ_MRVC01000000
VP50	CFSAN026729	Maryland	USA	2013	Clinical	NZ_MRVB01000000
VP30	CFSAN006130	Maryland	USA	2013	Clinical	JNTV02000000
VP46	CFSAN007462	Maryland	USA	2013	Clinical	JNTU02000000
VpG-8	CA_M12X02764	California	USA	2012	Clinical	LHRA00000000
VpG-7	CA_M12X02763	California	USA	2012	Clinical	LHRB00000000
3324	3324	Washington	USA	2007	Clinical	NZ_AOPA00000000
K1461	K1461	Massachusetts	USA	2004	Clinical	NZ_JMMO01000000
VpG-11	CDCK5629	Georgia	USA	2007	Clinical	LHQY00000000
MAVP-36	MAVP-36	Massachusetts	USA	2013	Clinical	NZ_LBHE00000000
VP32	CFSAN006131	Maryland	USA	2013	Clinical	JNTN02000000
CFSAN022332	G31	Galicia	Spain	2012	Clinical	LHRT00000000
CFSAN022335	G36	Galicia	Spain	2012	Clinical	LHRV00000000
CFSAN022334	G37	Galicia	Spain	2012	Clinical	LHRU00000000
CFSAN022331	G30	Galicia	Spain	2012	Clinical	LHRS00000000
CFSAN022336	G35	Galicia	Spain	2012	Clinical	LHRW00000000
CFSAN022330	G25	Galicia	Spain	2012	Clinical	LHRR00000000
CFSAN062371	3.369–15	Lima	Peru	2011	Environmental	SRR8103880
CFSAN062273	1.210–16	Lima	Peru	2016	Clinical	SRR8103884
10–4298	10–4298	British Columbia	Canada	2001	Clinical	JXUZ00000000
CFSAN018774	CDCA8962	Massachusetts	USA	2008	Clinical	LHRO00000000
A1EZ679	A1EZ679	British Columbia	Canada	2001	Clinical	NZ_LRSZ00000000
A2EZ715	A2EZ715	British Columbia	Canada	2002	Clinical	NZ_LRFQ00000000
10–4293	10–4293	British Columbia	Canada	2002	Clinical	JXVA00000000
A1EZ919	A1EZ919	British Columbia	Canada	2001	Clinical	NZ_LNTX00000000
A2EZ523	A2EZ523	British Columbia	Canada	2002	Clinical	NZ_LRTA00000000
10329	10329	Washington	USA	1998	Clinical	JWSS00000000
10–4303	10–4303	British Columbia	Canada	2000	Clinical	JXUY00000000
A0EZ608	A0EZ608	British Columbia	Canada	2000	Clinical	NZ_LRFM00000000
EN9901310	EN9901310	Washington	USA	1999	Clinical	NZ_AOPL00000000
10290	CFSAN001613	Washington	USA	1997	Clinical	JNUF02000000
O29–1-b	029–1(b)	Washington	USA	1997	Environmental	JNTW02000000
A5Z905	A5Z905	British Columbia	Canada	2005	Clinical	NZ_LQCU00000000
10296	10296	Washington	USA	1997	Clinical	AYSP01000000
EN9701173	EN9701173	Washington	USA	1997	Clinical	NZ_AOPK00000000
NY-3483	NY-3483	New York	USA	1998	Clinical	JNUC02000000
48057	48057	Washington	USA	1990	Clinical	JNTX02000000
VP43–1A	CFSAN001621	Washington	USA	1992	Environmental	LHQV00000000
JJ21–1C	CFSAN001615	Washington	USA	1990	Environmental	LHPD00000000
48291	48291	Washington	USA	1990	Clinical	JNUA02000000
F11–3A	F11–3A	Washington	USA	1988	Environmental	JNUB02000000

*ST, sequence type.
